# Age-specific differences in breast cancer treatment between screen-detected and non-screen-detected breast cancers in women aged 40–74 years at diagnosis in Sweden 2008–2017

**DOI:** 10.2340/1651-226X.2024.40200

**Published:** 2024-07-05

**Authors:** Håkan Jonsson, Anne Andersson, Zheng Mao, Lennarth Nyström

**Affiliations:** a Department of Radiation Sciences, Oncology, Umeå University, Umeå, Sweden; b Department of Epidemiology and Global Health, Umeå University, Umeå, Sweden

**Keywords:** Breast cancer, screening programme, mammography, surgery, chemotherapy, radiotherapy, endocrine therapy, antibody therapy

## Abstract

**Background and purpose:**

We have recently demonstrated that screen-detected invasive breast cancers had more favourable tumour characteristics than non-screen-detected. The objective of the study was to analyse differences in breast cancer treatment between screen-detected and non-screen-detected cases by age at diagnosis, with and without adjustment for tumour (T) and nodal (N) status, within a nationwide, population-based mammography screening programme utilising register data.

**Material and methods:**

Data spanning 2008–2017 were collected from the National Quality Register for Breast Cancer. Multivariable logistic regression analysis was used to estimate odds ratios and 95% confidence intervals for treatment disparities between screen-detected and non-screen-detected breast cancer.

**Results:**

Among 46,481 women diagnosed with invasive breast cancer aged 40–74 and invited for mammography screening, significant differences in treatment were observed. Screen-detected cases showed higher likelihoods of partial mastectomy compared to mastectomy, endocrine therapy, and radiotherapy, whereas chemotherapy and antibody therapy were less likely compared to non-screen-detected cases. However, when adjusting for surgery type, screen-detected cases showed lower likelihoods of radiotherapy. Age at diagnosis significantly influenced treatment odds ratios, with interactions observed for all treatments except radiotherapy adjusted for surgery. Differences increased with age, except for endocrine therapy. Radiotherapy adjusted for surgery type showed no age-related interaction. Adjusting for T and N did not alter these patterns.

**Interpretation:**

In general, screen-detected cases received less aggressive treatment, such as mastectomy, chemotherapy, and antibody therapy, compared to non-screen-detected cases. Disparities increased with age, except for endocrine therapy and radiotherapy adjusted for surgery. Differences persisted after adjusting for T and N, suggesting that these factors cannot solely explain the results.

## Introduction

According to the national cancer and cause of death register in Sweden, in 2021, 8,619 women were diagnosed with breast cancer and 1,326 women died with breast cancer as the underlying cause of death [1].

Overviews and meta-analysis of the randomised controlled trials on mammography screening have shown that breast cancer screening with mammography can reduce the mortality from breast cancer for women aged 50–69 years with 20%–25% [2–4].

In 1986, the National Board of Health and Welfare in Sweden recommended the county councils to invite women aged 40–74 years to mammography screening. The programme was fully implemented in all 21 counties in 1997 for the 50–69-year age group and in 2012 for the 40–49 and 70–74-year age groups.

Several small studies in Finland, Italy, Japan, Sweden and the UK have compared treatment modalities between screen-detected and non-screen-detected breast cancer cases. Screen-detected women had a higher proportion of partial mastectomy (conservative surgery) (three studies) [5–7], a lower proportion of chemotherapy (three studies) [6–8], a higher proportion of endocrine therapy (one study) [6], and a lower proportion of adjuvant systemic therapy (two studies) [9, 10]. However, in all, except two, studies with recent data [5, 6], all or a great part of the cases were treated in the 1990s. None of the studies compared the treatment in relation to age at diagnosis.

The aim of screening is to reduce the risk of breast cancer deaths. Early detection of cancer through screening may result in the tumour being detected at an earlier stage, where it may be less aggressive and easier to treat effectively. We have recently demonstrated that screen-detected invasive breast cancers had more favourable tumour characteristics than non-screen-detected cases after adjusting for age, calendar year and county of diagnosis and even after adjusting for T and N [11]. The trend towards favourable tumour characteristics was less pronounced in the 40–49 age group compared to the 50–74-year age group, except for the oestrogen and progesterone receptors. The aim of this study was to analyse differences in breast cancer treatment between screen-detected and non-screen-detected cases by age at diagnosis, with and without adjustment for tumour (T) and nodal (N) status, within a nationwide, population-based mammography screening programme using register data.

## Material and methods

### Data retrieval

Data on all female breast cancers diagnosed during 2008–2017 were retrieved from the National Quality Register for Breast Cancer (NKBC), with a 99.8% coverage (2008–2017) [12, 13]. In the NKBC, only one tumour, regardless of invasive or in situ, can be recorded per breast. Except for individual information on whether the cancer was screen-detected or non-screen-detected (interval cancer or cancer in non-participants), tumour characteristics, age, date, and residence (county) at diagnosis, the register also contains information on planned and given treatment. Data were also linked to the National Cancer Register mainly to get information if a woman also had a breast cancer before 2008 or after 2017.

### Treatment

The studied treatments were surgery, chemotherapy, radiotherapy, endocrine therapy and antibody therapy. However, based on treatment guidelines, endocrine therapy is only considered if the tumour is ER+, and antibody therapy is considered only if the tumour is HER2+. The treatments were compared between screen-detected and non-screen-detected cases. However, since most patients had surgery, the comparison was made between partial mastectomy and mastectomy, not between surgery and no surgery. Both planned and administered treatment are recorded. However, due to a substantial amount of missing data, we only used planned treatment. Since radiotherapy has a strong relationship with surgery (adjuvant treatment recommended after partial mastectomy), we also studied radiotherapy adjusted for type of surgery. Cases with a second breast cancer occurring within a year after the first breast cancer were excluded to prevent the selected treatment from being influenced by the second occurrence.

In Sweden, invitation to screening for women aged 50–69 has been ongoing since the 1990s; however, for the age groups 40–49 and 70–74, screening was introduced later in several counties. To ensure that all included cases had been invited to screening before the diagnosis, we excluded all cases 40–49 and/or 70–74 years in these counties the first 2 years after the start of screening.

The average mammography screening attendance rate in Sweden during 2017–2018 was 81% [14].

### Statistical methods

Multivariable logistic regression analysis was used to estimate the odds ratio (OR) and 95% confidence intervals (CI) for screen-detected versus non-screen-detected cancers for treatment modalities. For surgery, the OR was calculated for the likelihood of partial mastectomy. The analysis was adjusted for age group, calendar year and county at diagnosis (model I) and further also for tumour size and nodal status (model II). Cases with no surgery were rare (*n* = 92) and excluded from the statistical analysis of surgery. In the logistic regression models, cases with missing data were excluded.

Data were also analysed by age group at diagnosis to assess if the ORs varied by age, that is interaction. Interaction means that the ORs for a certain treatment differed by age and was tested using the likelihood ratio test. A *p* < 0.05 was considered statistically significant as well as 95% CI for OR which did not cover one.

The statistical software R (version 4.2.2) was used for all calculations [15].

## Results

The study population comprised all women diagnosed with breast cancer during 2008–2017 at the age of 40–74 years. Women with more than one breast cancer were excluded if the first cancer did not meet these criteria or if the second cancer was diagnosed less than a year after the first. For women with multiple breast cancers, all second cancers were excluded. Women aged 40–49 and 70–74 years at diagnosis, who were diagnosed earlier than 2 years after screening began, were excluded. Cases with missing data on detection mode or the pathological anatomical diagnosis (PAD), as well as non-invasive cases were also excluded. In the final dataset, 46,481 cases of invasive breast cancer remained for analysis. Table 1 shows the number of screen-detected and non-screen-detected invasive breast cancer cases by age group at diagnosis and treatment.

**Table 1:** Number of screen-detected and non-screen-detected invasive breast cancer cases by treatment and age group at diagnosis.

		Age group at diagnosis				
		40–49	50–59	60–69	70–74	40–74
Screen-detected	No	3,564	5,376	6,190	2,089	17,219
	Yes	3,237	7,622	1,3375	5,028	29,262
Surgery	Partial mastectomy	3,808	8,339	13,226	4,565	29,938
	Mastectomy	2,815	4,269	5,711	2,264	15,059
	No surgery	9	32	29	22	92
	Missing	169	358	599	266	1,392
Chemotherapy	No	2,821	6,571	11,852	4,873	26,117
	Yes	3,597	5,642	6,352	1,647	17,238
	Missing	383	785	1,361	597	3,126
Radiotherapy	No	1,204	1,974	3,302	1,575	8,055
	Yes	5,271	10,335	15,057	5,022	35,685
	Missing	326	689	1,206	520	2,741
Endocrine therapy	No	1,453	2,916	4,081	1,391	9,841
	Yes	4,981	9,321	14,203	5,176	33,681
	Missing	367	761	1,281	550	2,959
Antibody therapy	No	5,271	10,275	16,102	5,919	37,567
	Yes	999	1,692	1,746	505	4,942
	Missing	531	1,031	1,717	693	3,972
Total number		6,801	12,998	19,565	7,117	46,481

The percentage of invasive breast cancer cases receiving partial mastectomy, endocrine treatment, and radiotherapy was higher for screen-detected cases than for non-screen-detected cases in all age groups, except for women aged 70–74 years at diagnosis receiving endocrine therapy. In contrast, the percentage receiving chemotherapy and antibody treatment was lower for screen-detected cases compared to non-screen-detected cases (Supplementary Table). The largest differences were seen in partial mastectomy and chemotherapy.

Logistic regression analysis showed that the likelihood of receiving partial mastectomy, radiotherapy and endocrine therapy was significantly higher and the likelihood of receiving chemotherapy and radiotherapy adjusted for type of surgery and antibody therapy was significantly lower in screen-detected vs. non-screen-detected. Adjustment for age, calendar year and county (model I) only changed the ORs marginally. Further adjustment for T and N (model II) reduced the magnitude of the ORs (closer to 1) for partial mastectomy, chemotherapy, radiotherapy adjusted for surgery and antibody therapy (Table 2). When radiotherapy was adjusted for type of surgery, the OR changed from 1.4 to 0.59.

**Table 2:** Odds ratio (OR) and 95% confidence intervals (CI) of treatment of screen-detected vs non-screen-detected invasive breast cancer in women aged 40–74 years at diagnosis. Univariate analysis and multivariable analysis adjusted for age at diagnosis, calendar time and county of residence (model I) and age at diagnosis, calendar time, county of residence, T and N (model II).

Treatment	Univariate analysis		Adjusted model I		Adjusted model II	
	OR	CI	OR	CI	OR	CI
Surgery (Partial mastectomy)	2.56	2.45–2.66	2.54	2.44–2.65	1.78	1.70-1.87
Chemotherapy	0.43	0.42–0.45	0.47	0.45–0.49	0.70	0.67–0.74
Radiotherapy	1.43	1.36–1.50	1.50	1.43–1.58	1.54	1.46–1.63
Radiotherapy adjusted for surgery	0.59	0.56–0.63	0.63	0.59–0.67	0.89	0.82–0.96
Endocrine therapy	1.14	1.09–1.20	1.19	1.13–1.24	1.32	1.26–1.39
Antibody therapy	0.58	0.55–0.61	0.62	0.58–0.66	0.71	0.67–0.76

OR: odds ratio; CI: confidence intervals.

When analysing the age-specific ORs adjusted for year of diagnosis and county, all treatments, except radiotherapy adjusted for surgery, showed statistically significant interactions; that is, the ORs were not equal across age (Table 3). The ORs moved further away from 1 by increasing age, except for endocrine therapy where OR decreased from 1.5 for women aged 40–49 years to 0.93 for the age group 70–74. Furthermore, adjusting for both T and N did not alter the pattern, although the ORs approached closer to 1. All interactions, except radiotherapy adjusted for surgery, were statistically significant (Table 4).

**Table 3:** Odds ratio (OR) and 95% confidence intervals (CI) for treatment of screen-detected vs non-screen-detected invasive breast cancer cases by age group at diagnosis adjusted for calendar year and county of residence. P-values for test of interaction between treatment and age group at diagnosis.

Treatment	40–49		50–59		60–69		70–74		*p*
	OR	CI	OR	CI	OR	CI	OR	CI	
Surgery (Partial mastectomy)	1.87	1.69–2.06	2.31	2.14–2.50	2.91	2.73–3.12	3.11	2.78–3.47	<0.001
Chemotherapy	0.64	0.58–0.71	0.48	0.44–0.51	0.42	0.39–0.45	0.43	0.38–0.48	<0.001
Radiotherapy	1.21	1.07–1.37	1.43	1.29–1.57	1.64	1.51–1.77	1.62	1.44–1.83	0.001
Radiotherapy adjusted for surgery	0.70	0.60–0.81	0.67	0.59–0.75	0.61	0.55–0.67	0.54	0.46–0.63	0.07
Endocrine therapy	1.45	1.29–1.63	1.33	1.22–1.45	1.08	1.00–1.17	0.93	0.81–1.06	<0.001
Antibody therapy	0.76	0.66–0.87	0.66	0.59–0.73	0.55	0.50–0.61	0.52	0.43–0.63	<0.001

OR: odds ratio; CI: confidence intervals.

**Table 4:** Odds ratio (OR) and 95% confidence intervals (CI) for treatments for screen-detected vs non-screen-detected invasive breast cancer cases by age group at diagnosis adjusted for calendar year, county of residence, T and N status. P-values for test of interaction between treatment and age group.

Treatment	40–49		50–59		60–69		70–74		*p*
	OR	CI	OR	CI	OR	CI	OR	CI	
Surgery (Partial mastectomy)	1.45	1.30–1.62	1.62	1.49–1.76	1.97	1.83–2.12	2.11	1.87–2.39	<0.001
Chemotherapy	0.84	0.75–0.94	0.69	0.63–0.75	0.66	0.61–0.71	0.70	0.61–0.80	0.005
Radiotherapy	1.25	1.09-1.42	1.46	1.32–1.62	1.70	1.56–1.84	1.65	1.45-1.87	<0.001
Radiotherapy adjusted for surgery	0.85	0.71–1.03	0.92	0.80–1.06	0.93	0.82–1.05	0.77	0.64–0.93	0.34
Endocrine therapy	1.61	1.42–1.82	1.48	1.36–1.62	1.21	1.12–1.31	1.02	0.89–1.17	<0.001
Antibody therapy	0.84	0.73–0.96	0.75	0.68–0.84	0.65	0.58–0.72	0.62	0.51–0.75	0.008

OR: odds ratio; CI: confidence intervals.

## Discussion

This study shows that radiotherapy, endocrine therapy and partial mastectomy are more frequently performed, while chemotherapy and antibody therapy are less frequently performed in screen-detected breast cancer cases compared to non-screen-detected cases. However, when adjusted for type of surgery, also radiotherapy was less common in screen-detected cases, most likely because radiotherapy is recommended after a partial mastectomy but not after a mastectomy if N-; screen-detected cases had a higher proportion of partial mastectomy. The results also showed statistically significant different ORs by age (interaction) for all studied treatments, except radiotherapy adjusted for surgery. Except for endocrine therapy, all differences between screen-detected and non-screen-detected breast cancer cases increased by age. After adjustment for T and N, the differences between screen-detected and non-screen-detected cases remained statistically significant although somewhat smaller except for endocrine therapy where the differences increased.

The findings regarding partial mastectomy, chemotherapy, and endocrine therapy are consistent with all published studies. Radiotherapy was analysed in one study, but no significant result was found [7]. No other study adjusted for T and N.

Data on the breast cancer cases were retrieved from a high-quality nationwide register, with almost 100% coverage. The large size of the study resulted in narrow CI and possibilities to also analyse interaction with age at diagnosis. The ORs were calculated with adjustments for calendar year, age and county at diagnosis. Radiotherapy was analysed with and without adjustment for type of surgery. All analyses were made with and without adjustment for T and N which facilitates different interpretations.

Data in NKBC have been validated by all six Regional Cancer Centres. In total, 800 breast cancer cases diagnosed in 2013 were randomly selected and a 5% disagreement in mode of detection (screen-detected/non-screen-detected) was found [16]. The misclassification might bias the differences in the proportion of screen-detected and non-screen-detected cases, but it is probably not related to treatment, that is no differential misclassification.

Information on planned oncologic therapy was chosen as the coverage in the register was better than for given treatment. In a study of 970 women, aged 20–63 years living in Stockholm County, Sweden, who had undergone breast cancer surgery, the agreement between recommended and actual initiated treatment was found to be between 93.9% and 96.1% for radiotherapy, chemotherapy and endocrine therapy [17].

The study did not include neoadjuvant treatment. Interactive statistics from NKBC revealed that neoadjuvant endocrine treatment accounted for less than 1 per 1,000 cases [13]. In contrast, neoadjuvant chemotherapy showed a more prevalent and increasing trend, rising from 4.1% in 2012–2013 to 7.1% in 2016–2017.

Non-screen-detected cases were constituted of a mix of interval cancers and non-participants in screening with unknown proportions. The proportion of interval cancer in non-screen detected cases was estimated to be 67% using the same method as in Jonsson et al. [11], assuming that the participation rate is 81%.

In the age group 40–49, women aged 40–41 might have received a breast cancer diagnosis before their initial screening invitation. Thus, the age group 40–41 may deviate from the rest of the cases in the age group. As a sensitivity analysis, we examined the outcomes by excluding women at age 40–41 from the 40–49 age group, and the deviations from the aforementioned results were minor. The most notable differences were observed when adjusted for calendar year, county of residence, and T and N status. In the age group 42–49, the OR for radiotherapy, adjusted for type of surgery, was 0.81 (95% CI: 0.66–0.99), and for antibody therapy, it was 0.78 (95% CI: 0.67–0.90), compared to ORs of 0.85 and 0.84 for the 40–49 age group (Table 4). Additionally, the interaction between age and antibody therapy became non-significant (*p* = 0.06).

## Conclusions

Substantial differences in treatment between screen-detected and non-screen-detected breast cancer were found. Screen-detected breast cancer got more partial mastectomy compared to mastectomy, radiotherapy and endocrine therapy and less chemotherapy, antibody treatment and radiotherapy adjusted for surgery compared to cases invited, but non-screen-detected. Furthermore, the differences increased with increasing age. The only exception was endocrine treatment which also varied over age but with decreasing differences. The only treatment where no statistically significant differences between age groups could be seen was radiotherapy adjusted for surgery. All findings remained even after adjustment for tumour size and nodal status that is T and N. Differences in treatment of breast cancer between screen-detected and non-screen-detected cases cannot be explained only by size and nodal status. Thus, early detection of cancer through screening results in the tumour being detected at an earlier stage, leading to less aggressive treatment.

## Supplementary Material

Age-specific differences in breast cancer treatment between screen-detected and non-screen-detected breast cancers in women aged 40–74 years at diagnosis in Sweden 2008–2017

## Data Availability

The data cannot be shared publicly because the individual-level data contain potentially identifying and sensitive patient information and cannot be published due to legislation and ethical review restrictions (https://etikprovningsmyndigheten.se).

## References

[1] Statistics on new detected cancer cases 2021 (The National Board of Health and Welfare, in Swedish). 2022. [Cited date June 27, 2024] Available from: https://www.socialstyrelsen.se/globalassets/sharepoint-dokument/artikelkatalog/statistik/2022-12-8308.pdf

[2] Independent UK Panel on Breast Cancer Screening. The benefits and harms of breast cancer screening: an independent review. Lancet. 2012;380:1778–1786. 10.1016/S0140-6736(12)61611-0 23117178

[3] Nyström L, Rutqvist L, Wall S, Lindgren A, Lindqvist M, Rydén S, et al. Breast cancer screening with mammography: overview of Swedish randomised trials. Lancet. 1993;341:973–978. 10.1016/0140-6736(93)91067-V 8096941

[4] European guidelines on breast cancer screening and diagnosis (European Commission). 2023. [Cited date June 27, 2024] Available from: https://healthcare-quality.jrc.ec.europa.eu/ecibc/european-breast-cancer-guidelines

[5] Fancellu A, Sanna V, Sedda ML, Delrio D, Cottu P, Spanu A, et al. Benefits of organized mammographic screening programs in women aged 50 to 69 years: A surgical perspective. Clin Breast Cancer. 2019;19:e637–e642. 10.1016/j.clbc.2019.04.013 31130366

[6] Kobayashi N, Hikichi M, Ushimado K, Sugioka A, Kiriyama Y, Kuroda M, et al. Differences in subtype distribution between screen-detected and symptomatic invasive breast cancer and their impact on survival. Clin Transl Oncol. 2017;19:1232–1240. 10.1007/s12094-017-1660-z 28409323

[7] Olsson A, Borgquist S, Butt S, Zackrisson S, Landberg G, Manjer J. Tumour-related factors and prognosis in breast cancer detected by screening. Br J Surg Soc. 2012;99:78–87. 10.1002/bjs.7757 22068957

[8] Dawson SJ, Duffy SW, Blows FM, Driver KE, Provenzano E, LeQuesne J, et al. Molecular characteristics of screen-detected vs symptomatic breast cancers and their impact on survival. Br J Cancer. 2009;101:1338–1344. 10.1038/sj.bjc.6605317 19773756 PMC2768460

[9] Joensuu H, Lehtimaki T, Holli K, Elomaa L, Turpeenniemi-Hujanen T, Kataja V, et al. Risk for distant recurrence of breast cancer detected by mammography screening or other methods. JAMA. 2004;292:1064–1073. 10.1001/jama.292.9.1064 15339900

[10] Sihto H, Lundin J, Lehtimaki T, Sarlomo-Rikala M, Butzow R, Holli K, et al. Molecular subtypes of breast cancers detected in mammography screening and outside of screening. Clin Cancer Res. 2008;14:4103–4110. 10.1158/1078-0432.CCR-07-5003 18593987

[11] Jonsson H, Andersson A, Mao Z, Nystrom L. Age-specific differences in tumour characteristics between screen-detected and non-screen-detected breast cancers in women aged 40–74 at diagnosis in Sweden from 2008 to 2017. J Med Screening. 2024 March 7 [Available online]. 10.1177/09691413241237616 PMC11526418 38454634

[12] National quality register for breast cancer (Regional Cancer Centers in collaboration, in Swedish). 2023. [Cited date June 27, 2024] Available from: https://cancercentrum.se/samverkan/cancerdiagnoser/brost/kvalitetsregister/

[13] Interactive report: National quality register for breast cancer (In Swedish). 2023. [Cited date June 27, 2024] Available from: https://statistik.incanet.se/brostcancer/

[14] Broman C, Edbom T. National evaluation – breast cancer screening with mammography (In Swedish). The National Board of Health and Welfare, Stockholm; 2022.

[15] R: A language and environment for statistical computing. Vienna, Austria: R Foundation for Statistical Computing; 2022. [Cited date June 27, 2024] Available from: https://www.R-project.org/

[16] Validation of National quality register for breast cancer (Regional Cancer Centers in collaboration, in Swedish). 2015. [Cited date June 27, 2024] Available from: https://cancercentrum.se/globalassets/cancerdiagnoser/brost/kvalitetsregister/rapport_master_validering_brostregister2015-10-14.pdf

[17] Wennman-Larsen A, Nilsson MI, Saboonchi F, Olsson M, Alexanderson K, Fornander T, et al. Can breast cancer register data on recommended adjuvant treatment be used as a proxy for actually given treatment? Eur J Oncol Nurs. 2016;22:1–7. 10.1016/j.ejon.2016.02.010 27179887

