# Supplementary material for: Age-specific differences in breast cancer treatment between screen-detected and non-screen-detected breast cancers in women aged 40–74 years at diagnosis in Sweden 2008–2017
Source: Acta Oncol. 2024 Jul 5;63:40200. doi: 10.2340/1651-226X.2024.40200 (PMC11332461; doi:10.2340/1651-226X.2024.40200)
Supplement: Age-specific differences in breast cancer treatment between screen-detected and non-screen-detected breast cancers in women aged 40–74 years at diagnosis in Sweden 2008–2017 [file AO-63-40200-s1.pdf]

Supplementary material has been published as submitted. It has not been copyedited, or typeset by Acta Oncologica

Supplementary Table. Percentage (%) and total number (No) of screen-detected (SD) and non-screen-detected (NSD) invasive breast cancer cases by type of treatment and age group at diagnosis. Missing data excluded.

Treatment			Age group at diagnosis				
			40-49	50-59	60-69	70-74	40-74
Partial mastectomy	SD	%	65	73	77	74	74
		No	3201	7568	13259	4942	28970
	NSD	%	51	55	54	48	53
		No	3422	5040	5678	1887	16027
Chemotherapy	SD	%	50	39	29	21	33
		No	3051	7254	12651	4691	27647
	NSD	%	61	57	49	37	53
		No	3367	4959	5553	1829	15708
Radiotherapy	SD	%	83	86	84	79	84
		No	3088	7311	12765	4743	27907
	NSD	%	80	81	77	70	78
		No	3387	4998	5594	1854	15833
Endocrine therapy	SD	%	81	78	78	78	78
		No	3067	7276	12709	4718	27770
	NSD	%	75	74	77	80	76
		No	3367	4961	5575	1849	15752
Antibody therapy	SD	%	14	12	8.1	6.4	10
		No	3004	7146	12492	4628	27270
	NSD	%	18	14	17	12	15
		No	3266	4821	5356	1796	15239
